# Clonal integration benefits an invader in heterogeneous environments with reciprocal patchiness of resources, but not its native congener

**DOI:** 10.3389/fpls.2022.1080674

**Published:** 2022-12-01

**Authors:** Xiao-Mei Zhang, Lin-Xuan He, Xiao Xiao, Jing-Pin Lei, Min Tang, Ning-Fei Lei, Fei-Hai Yu, Jin-Song Chen

**Affiliations:** ^1^ Zhejiang Provincial Key Laboratory of Plant Evolutionary Ecology and Conservation, Institute of Wetland Ecology & Clone Ecology, Taizhou University, Taizhou, China; ^2^ College of Life Science, Sichuan Normal University, Chengdu, China; ^3^ Research Institute of Forestry, Chinese Academy of Forestry, Beijing, China; ^4^ College of Ecology and Environment, Chengdu University of Technology, Chengdu, China

**Keywords:** clonal functional trait, complementary patches, division of labor, invasiveness, physiological integration, resource sharing

## Abstract

Many of the world’s most invasive plants are clonal, and clonal functional traits are suggested to contribute to their invasiveness. Clonal integration is one of the most important clonal functional traits, but it is still unclear whether clonal integration can benefit invasive alien clonal plants more than native ones in heterogeneous environments with reciprocal patchiness of resources and whether invasive plants show a higher capacity of division of labor than native ones in such environments. We grew connected (allowing clonal integration) and disconnected (preventing clonal integration) ramet pairs of an invasive plant *Wedelia trilobata* and its occurring native congener *W. chinensis* in the environment consisting of reciprocal patches of light and soil nutrients (i.e., a high-light but low-nutrient patch and a low-light but high-nutrient patch). Clonal integration greatly promoted the growth of the invasive species, but had no significant effect on the native one. Both invasive and native species showed division of labor in terms of morphology, biomass allocation, and/or photosynthetic physiology, but the capacity of labor division did not differ between the invasive and the native species. We conclude that in heterogeneous environments consisting of reciprocal patches of resources, which are common in nature, clonal integration can confer invasive plants a competitive advantage over natives, but this difference is not related to their capacity of labor division. This study highlights the importance of clonal integration for plants in heterogeneous environments and suggests that clonal integration can contribute to the invasion success of alien clonal plants.

## Introduction

Invasive alien plants can severely threaten biodiversity conservation, ecosystem function and human health (Lodge, 1993; Feng et al., 2007a; Vilà et al., 2011). Numerous studies have been conducted to assess functional traits that can contribute to the invasion success of alien plants (Lodge, 1993; Alpert et al., 2000; Levine et al., 2003; Roiloa et al., 2016; He et al., 2021). An emerging pattern is that many of the world’s most invasive plant species are able to regenerate clonally (i.e., asexually) and thus have unique clonal life-history traits (McDowell, 2002; van Kleunen et al., 2002; Liu et al., 2006; Song et al., 2013; Wang et al., 2017). Thus, it is likely that these clonal traits have played a key role in the successful invasion of alien clonal plants into native plant communities (Kolar and Lodge, 2001; Song et al., 2013; Wang et al., 2017; Wang et al., 2022).

The environment that plants face is commonly not spatially uniform, but heterogeneous (Hutchings and Wijesinghe, 1997; Ikegami et al., 2008; Keser et al., 2014). Spatial heterogeneity in resource supply makes resource capture more costly and challenging for sessile organisms (Magyar et al., 2007; Ikegami et al., 2008; Wang et al., 2017; Li et al., 2018; Xue et al., 2020). However, clonal growth allows many clonal plants to produce interconnected ramets that are situated in patches (microsites) of different resource availability (Price and Marshall, 1999; Roiloa and Retuerto, 2006; Ikegami et al., 2008; You et al., 2016; Xue et al., 2020). *Via* clonal integration, interconnected ramets can transfer and share resources so that ramets growing in low-quality resource patches can get support from ramets growing in high-quality resource patches (Roiloa et al., 2010; Song et al., 2013; Wang et al., 2017). Such clonal integration can promote the performance of the whole clone because it commonly greatly benefits the ramets in the low-quality patches at no or low cost to the ramets in the high-quality patches (Song et al., 2013; You et al., 2016; Wang et al., 2021). Previous studies have shown that under such a type of heterogeneous environments (i.e., consisting of low- and high-quality patches) clonal integration can benefits invasive alien clonal plants more than their native congeners, suggesting that clonal integration can contribute to the invasion success of invasive alien clonal plants (Wang et al., 2017).

However, under some circumstances, environments can consist of reciprocal resource patches (Alpert and Stuefer, 1997; Stuefer, 1998; He et al., 2011; Zhang and Zhang, 2013). For instance, open patches with high light intensity are commonly short of soil water and nutrients and their adjacent shade patches with low light intensity are frequently abundant in soil water and nutrients (Alpert and Stuefer, 1997; Stuefer, 1998; Zhang and Zhang, 2013). Neither of these patches are favorable for individual plant (ramet) growth, but interconnected ramets that are situated in both types of patches are potentially beneficial because they can specialize to acquire locally abundant resources *via* changes in morphology, physiology and/or biomass allocation and mutually exchange acquired resources *via* clonal integration (Ikegami et al., 2008; Wang et al., 2011; Roiloa et al., 2014). For instance, ramets in the high-light but low-water/nutrient condition can increase leaf photosynthesis rate, leaf nitrogen content, leaf chlorophyll content, leaf size and/or biomass allocation to shoots and their connected ramets in the low-light but high-water/nutrient condition can increase water/nutrient uptake rate, specific root length and/or biomass allocation to roots, showing division of labor (Stueffer et al., 1996; Liao et al., 2003; Roiloa et al., 2007). Such clonal division of labor can greatly increase the efficiency of resource harvesting (Bloom et al., 1985; Stuefer, 1998; Freschet et al., 2018) and thus promote the performance of the whole clone (Friedman and Alpert, 1991; Stueffer et al., 1996; Roiloa et al., 2007; Roiloa et al., 2014; Lin et al., 2018).

While division of labor has been observed in a number of clonal plants and also in both invasive alien and native species under environments with reciprocal patchiness of resources (Friedman and Alpert, 1991; Roiloa et al., 2007; Ikegami et al., 2008; Wang et al., 2011; Roiloa et al., 2014; Huang et al., 2018), few studies have compared the ability of division of labor between invasive alien and native clonal plants. Comparing invasive alien species with their phylogenetically closely related native species (e.g., congeneric species) that occur in the same habitats is a powerful approach to understand the mechanisms underlying the invasion success of alien species (McDowell, 2002; Burns, 2004; van Kleunen et al., 2010; van Kleunen et al., 2014). Additionally, while clonal integration has been found to benefit invasive alien clonal plants more than their native congeners under heterogeneous environments consisting of low- and high-quality patches (Wang et al., 2017), it is still unknown whether clonal integration also benefits invasive alien clonal plants more than their native congeners under reciprocal patchiness of resources.

We grew connected (allowing clonal integration) and disconnected (preventing clonal integration) clonal fragments (each consisting of a developmentally older ramet and a developmentally younger ramet) of an invasive plant *Wedelia trilobata* and its congeneric native plant *Wedelia chinensis* under reciprocal patches of light and soil nutrients. Specifically, we tested the following hypotheses: (1) clonal integration increases the performance of both the invasive and the native plant under reciprocal patchiness of resources; (2) clonal integration benefits the invasive plant more than the native plant under such a type of heterogeneous environment; (3) both the invasive and the native plant show division of labor in terms of physiology, morphology and/or biomass allocation in the presence of clonal integration; (4) the ability of labor division is higher in the invasive than in the native plant.

## Materials and methods

### Plant material

*Wedelia trilobata* is a stoloniferous herb of the Asteraceae family and originates from the tropics of Central America (Thaman, 1999). It was introduced to China as an ornamental groundcover in 1970s, but has invaded many ecosystems in southern China after escaping from gardens (Si et al., 2014). Once established, this species can displace native species and forms mono-dominant community owing to its fast clonal growth (Song et al., 2010). *Wedelia trilobata* is listed as one of the 100 world’s worst invasive alien species (Lowe et al., 2000). Its congeneric species *Wedelia chinensis* is native to China, and has similar morphology and life history to *W. trilobata* (Song et al., 2010).

Offspring ramets of *W. trilobata* and *W. chinensis* used for this experiment originated from parent ramets collected in Haikou, Hainan Province, China (20^°^00^′^16^″^ N; 110^°^20^′^24^″^ E). These parent ramets were vegetatively propagated in a greenhouse at Jiangsu University in Zhenjiang, Jiangsu Province, China, and then some of their offspring ramets were vegetatively cultivated in a greenhouse at Sichuan Normal University in Chengdu, Sichuan Province, China (30^°^34^′^11^″^ N; 104^°^12^′^12^″^ E). In August 2021, 16 similar-sized clonal fragments of each of *W. trilobata* and *W. chinensis* were selected and each clonal fragment was comprised of two successive ramets (one developmentally older and one developmentally younger), each connected by a stolon internode. The two ramets of each clonal fragment were transplanted into two plastic pots (15 cm in diameter and 12 cm in height) filled with a 3:1:1 mixture of peat, vermiculite and perlite.

### Experimental design

**Figure 1:**
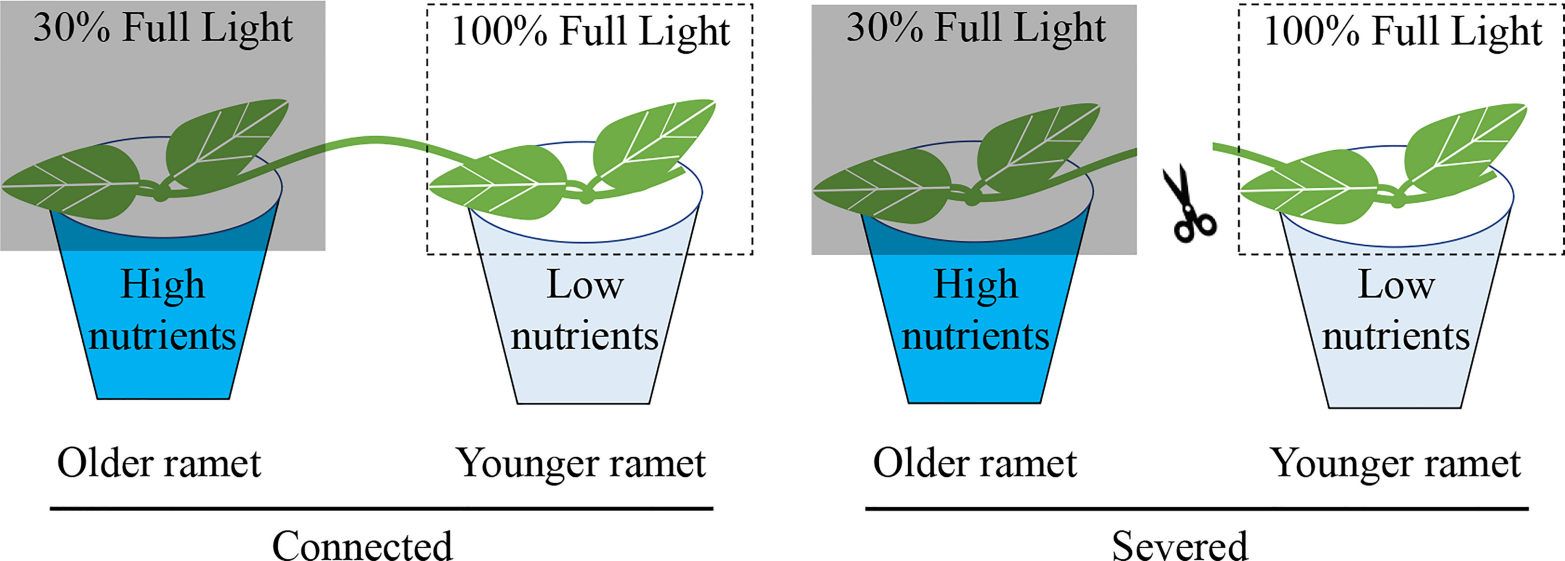
Schematic representation of the experiment design. Each clonal fragment consisted of a developmentally older and a developmentally younger ramet interconnected by a stolon internode. The older ramet was grown in a low-light (30% full light) and high-nutrient condition (with nutrient addition) and the younger ramet in a high-light (100% of full light) and low-nutrient condition (without nutrient addition). The stolon internode between the older and the younger ramet was either severed or kept intact (connected).

The experiment started one week after transplantation. The 16 clonal fragments of each species were randomly assigned to two treatments: (1) with clonal integration by keeping the stolon internode connecting the two ramets of each clonal fragment intact and (2) without clonal integration by severing the stolon internode connecting the two ramets of each clonal fragment (**Figure 1**). For each clonal fragment, the developmentally older ramet was grown under a high-nutrient but low-light condition and the developmentally younger ramet was grown under a low-nutrient but high-light condition. We did not include treatments in which the developmentally older ramet was grown under the low-nutrient but high-light condition and the developmentally younger ramet was grown under the high-nutrient but low-light condition because we expected that translocation of resources would be bidirectional, as reported before (e.g., Stueffer et al., 1996; Liao et al., 2003; Roiloa et al., 2007).

For the high-nutrient but low-light condition, the older ramet in each pot was supplied weekly with 100 mL concentrated nutrient solution after about 130-fold dilution (N: P: K=30:14:16; The Scotts Miracle-Gro Company) but shaded to 30% ambient light with black shading net. For the low-nutrient but high-light condition, the younger ramet in each pot was supplied with 100 mL of water and not shaded (i.e., under 100% ambient light).

The experiment lasted for 75 days and ended on 23 October 2021. During the experiment, new stolons generated from initial ramets were kept under the same light conditions as their parents and did not root. All pots were watered when the substrate surface became dry.

### Measurements

Before harvest, we measured net photosynthesis rate (P_n_) of each younger ramet by using the portable photosynthesis system (GFS-3000, Heinz Walz GmbH, Effeltrich, Germany). A fully expanded mature leaf from each younger ramet was selected and P_n_ was measured after 20 min of acclimation to the leaf cuvette microenvironment at a temperature of 28°C, photosynthetic photon flux density of 600 μmol·m^-2^·s^-1^ and CO_2_ concentration of 400 μmol·mol^-1^ (Wei et al., 2020). After P_n_ measurement, the leaf was harvested to determine chlorophyll content (Wei et al., 2019).

Each of younger and older ramet and their dependents was harvested and divided into leaves, stems, and roots. We scanned leaves and roots by using EPSON 11000XL Scanner, and the images were analyzed by Adobe Photoshop 2021 and WinRHIZO Pro 2016a to obtain leaf area and root traits. After that, all plant parts were dried to constant weight at 80°C to obtain biomass. We calculated specific leaf area (leaf area/leaf dry biomass), specific root length (root length/root dry biomass) and specific root surface area (root surface area/root dry biomass) (Merkl et al., 2005).

### Statistical analysis

Two-way ANOVA was used to investigate the effects of species, stolon connection and their interaction on photosynthetic characteristics (P_n_ and chlorophyll content), morphological characteristics (specific leaf area, area per leaf, specific root length, total root length, specific root surface area and total root surface area) and growth characteristics (total leaf area, total stem length, root biomass, stem biomass, leaf biomass, total biomass and root to shoot ratio). Following ANOVA, linear contrasts were used to compare the differences between the intact and severed treatments of each species. Prior to analysis, all data were checked for homogeneity of variance. All analyses were conducted with the SPSS 22.0 program (IBM Corporation, USA).

## Results

### Photosynthetic physiology of the younger ramet

**Figure 2:**
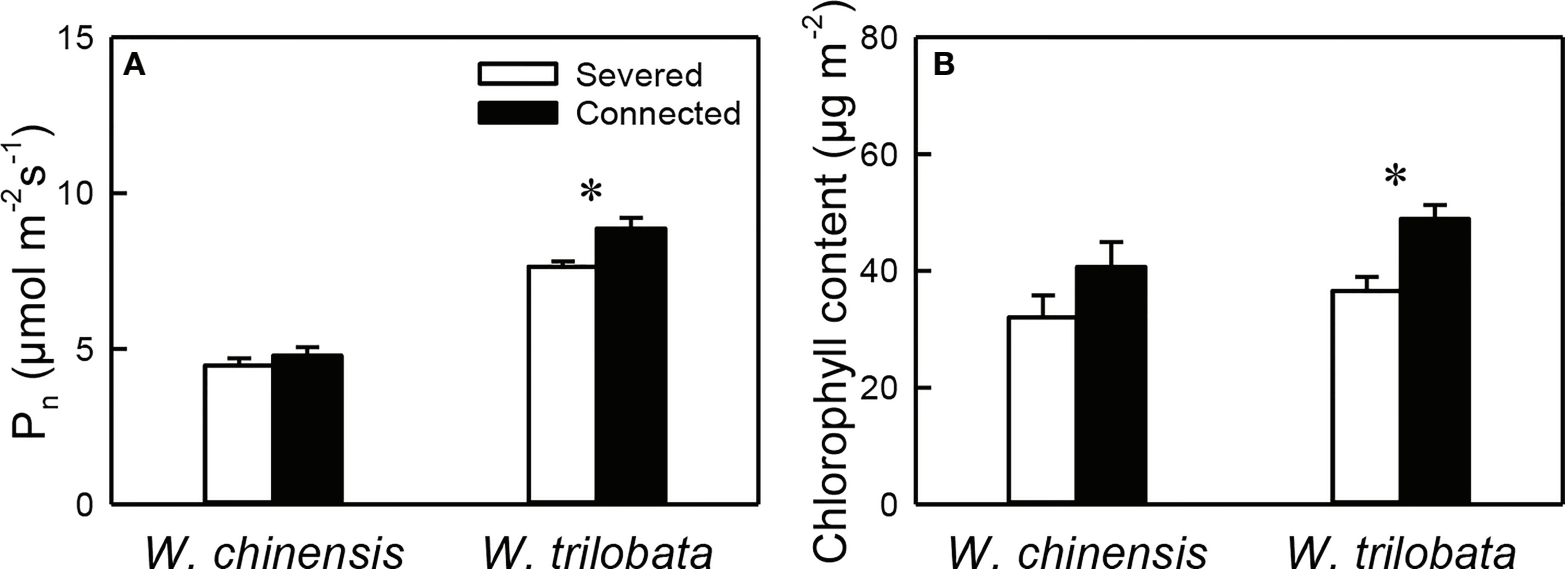
**(A)** Net photosynthesis rate (P_n_) and **(B)** chlorophyll content of the younger ramet of the invasive plant *Wedelia trilobata* and the native plant *W. chinensis*. Values are means ± SE. Symbols (^*^ *P* < 0.05) indicate the significant differences between the severed ramets and connected ramets within a species.

For the younger ramet, net photosynthesis rate (P_n_) and chlorophyll content were significantly higher in the connected than in the severed treatment in *W. trilobata*, but were not different between the two treatments in *W. chinensis* (**Figure 2**, **Appendix Table 1**). P_n_ of the younger ramet was also significantly higher in *W. trilobata* and in *W. chinensis* (**Figure 2A**, **Appendix Table 1**).

### Morphology and biomass allocation of the younger and the older ramet

**Figure 3:**
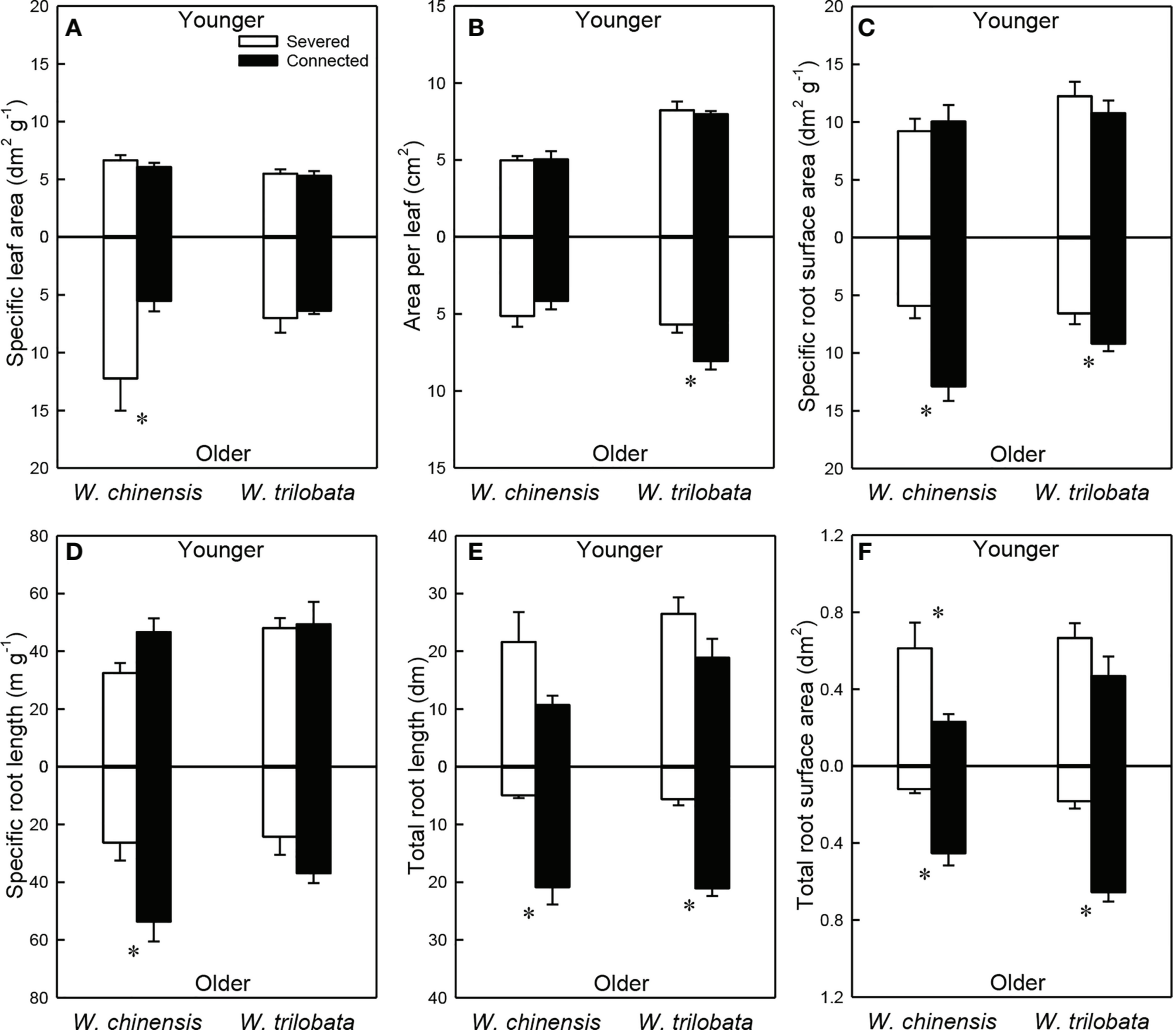
Specific leaf area **(A)**, area per leaf **(B)**, specific root surface area **(C)**, specific root length **(D)**, total root length **(E)** and total root surface area **(F)** of the younger ramet (above the x-axis) and the older ramet (below the x-axis) of the invasive plant *Wedelia trilobata* and the native plant *W. chinensis*. Values are means ± SE. Symbols (* *P* < 0.05) indicate the significant differences between the severed and connected ramets within a species.

For the younger ramet, stolon connection had no significant effect on specific leaf area, specific root length, specific root surface area and area per leaf in either species (**Figures 3A–D**; **Appendix Table 2A**). Total root length and total root surface area of the younger ramet were significantly or marginally significantly higher in the severed than in the connected treatment in *W. chinensis*, but not in *W. trilobata* (**Figures 3E, F**; **Appendix Table 2A**). The younger ramet of *W. trilobata* had larger mean leaf area and smaller specific leaf area than that of *W. chinensis* (**Figures 3A, B**; **Appendix Table 2A**).

For the older ramet of both *W. trilobata* and *W. chinensis*, specific root surface area, total root surface area and total root length were significantly greater in the connected than in the severed treatment (**Figures 3C, E, F**; **Appendix Table 2B**). Also, the connected older ramet produced significantly greater area per leaf than the severed older ramet in *W. trilobata* (**Figure 3B**), and produced significantly greater specific root length than the severed older ramet in *W. chinensis* (**Figure 3D**). However, specific leaf area of the older ramet and root surface area of the younger ramet showed the opposite pattern in *W. chinensis* (**Figures 3A, F**). The older ramet of *W. trilobata* had larger mean leaf area than that of *W. chinensis* (**Figure 3B**; **Appendix Table 2B**).

**Figure 4:**
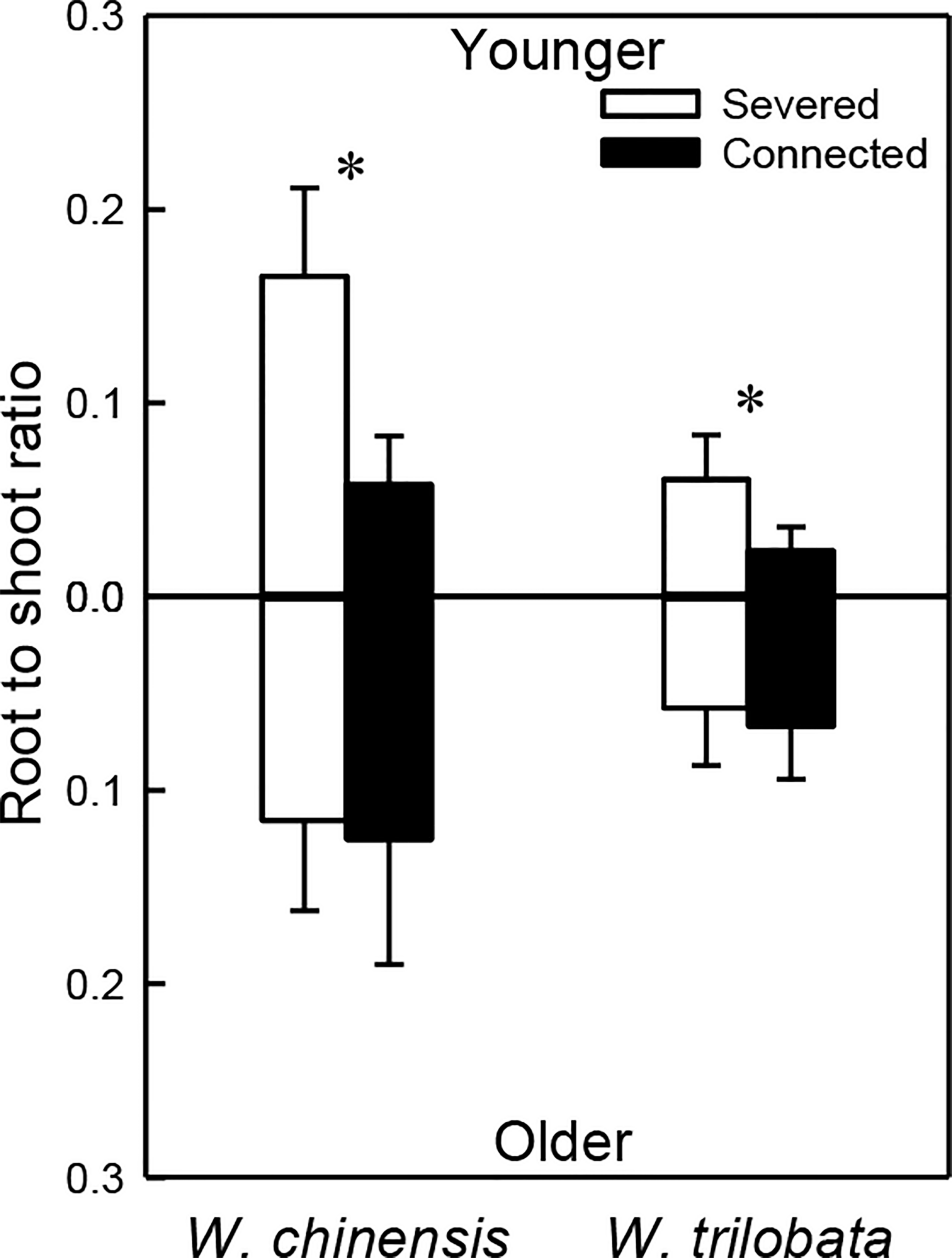
Root shoot ratio of the younger ramet (above the x-axis) and the older ramet (below the x-axis) of the invasive plant *Wedelia trilobata* and the native plant *W. chinensis*. Values are means ± SE. Symbols (* *P* < 0.05) indicate the significant differences between the severed ramets and connected ramets within a species.

For both *W. chinensis* and *W. trilobata*, stolon connection significantly decreased root to shoot ratio of the younger ramet, but had no effect on that of the older ramet (**Figure 4**; **Appendix Table 3**). Shoot to shoot ratio of both the younger and the older ramet was smaller in *W. trilobata* than in *W. chinensis* (**Figure 4**; **Appendix Table 3**).

### Growth of the younger and the older ramet

**Figure 5:**
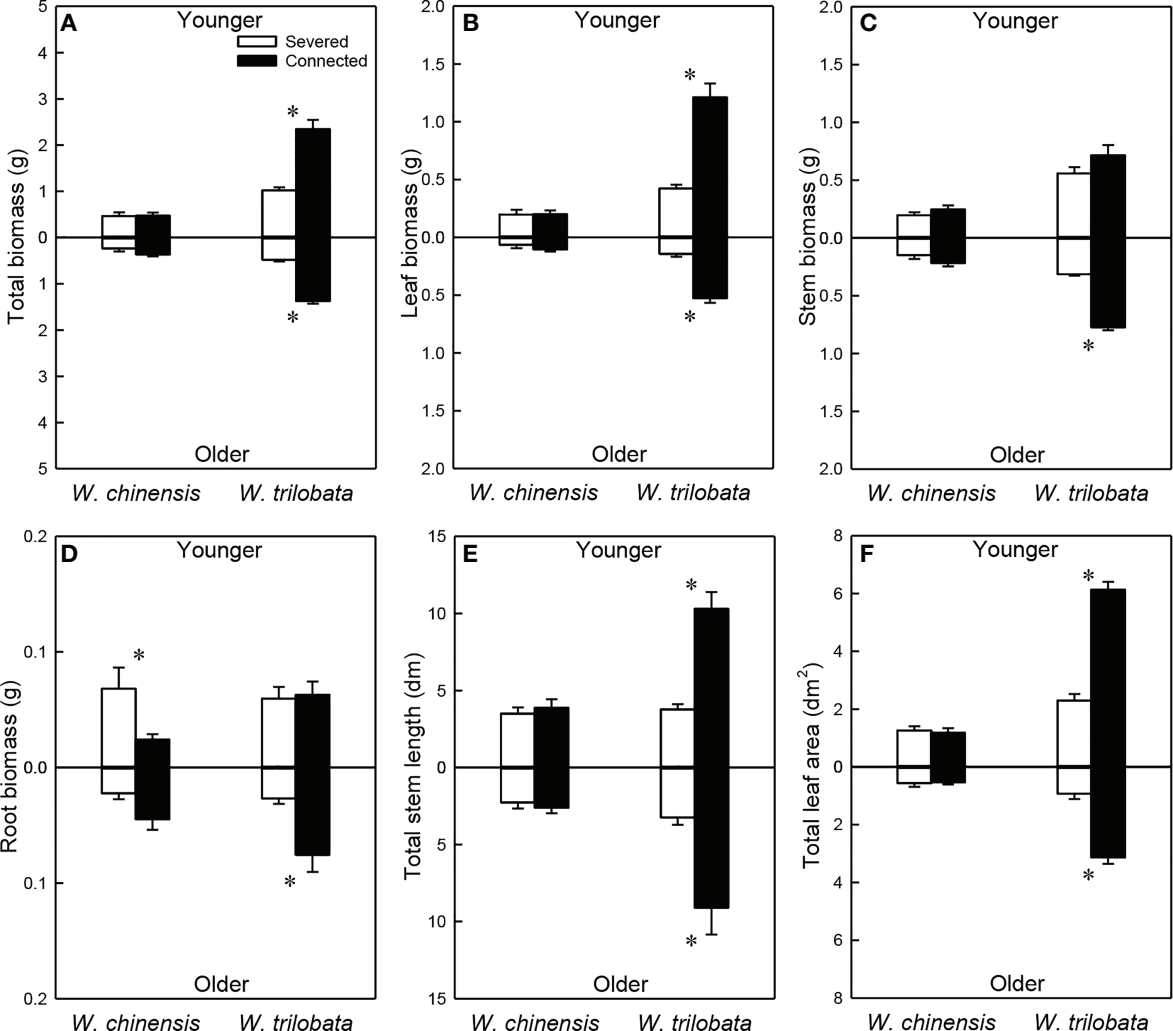
Total biomass **(A)**, leaf biomass **(B)**, stem biomass **(C)**, root biomass **(D)**, total stem length **(E)** and total leaf area **(F)** of the younger ramet (above the x-axis) and the older ramet (below the x-axis) of the invasive plant *Wedelia trilobata* and the native plant *W. chinensis*. Values are means ± SE. Symbols (* *P* < 0.05) indicate the significant differences between the severed ramets and connected ramets within a species.

For the younger ramet, stolon connection significantly increased total biomass, leaf biomass, total stem length and total leaf area in *W. trilobata*, but had no impact on these growth measures in *W. chinensis* (**Figures 5A, B, E, F**; **Appendix Table 3A**). For the younger ramet, stolon connection had no significant effect on stem biomass in either species (**Figure 5C**) or on root biomass in *W. trilobata*, but significantly decreased root biomass in *W. chinensis* (**Figure 5** **D**).

Stolon connection significantly increased all growth measures (total, leaf, stem and root biomass, total stolon length and total leaf area) of the older ramet in *W. trilobata*, but had no significant effect on any of these growth measures in *W. chinensis* (**Figure 5**; **Appendix Table 3B**). Total biomass, leaf biomass, stem biomass, total stem length and total leaf area of both the younger and the older ramets were larger in *W. trilobata* than in *W. chinensis* (**Figure 5**; **Appendix Table 3**).

### Growth of the clonal fragment

**Figure 6:**
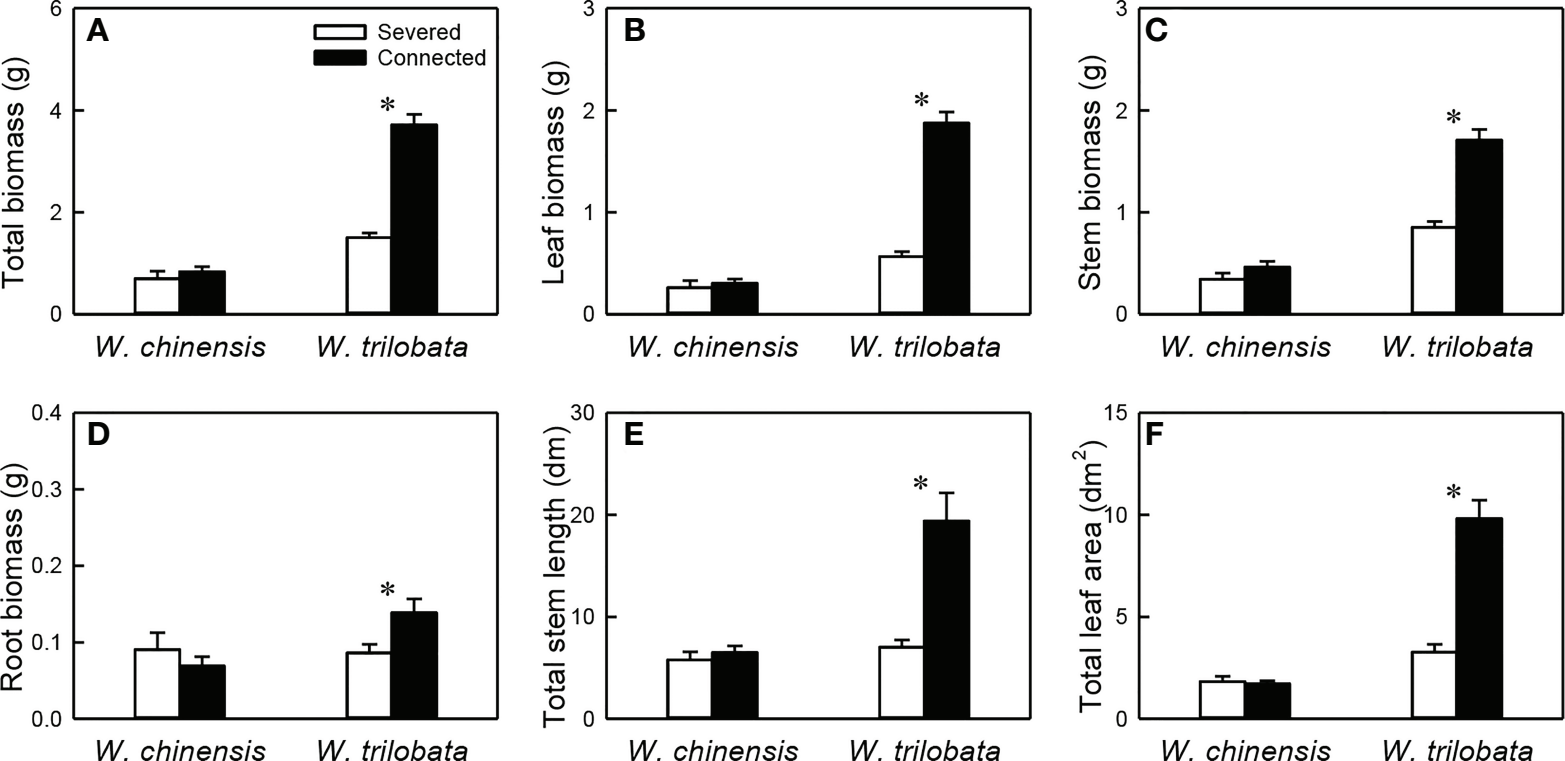
Total biomass **(A)**, leaf biomass **(B)**, stem biomass **(C)**, root biomass **(D)**, total stem length **(E)** and total leaf area **(F)** of the whole fragment of the invasive plant *Wedelia trilobata* and the native plant *W. chinensis*. Values are means ± SE. Symbols (* *P* < 0.05) indicate the significant differences between the severed ramets and connected ramets within a species.

For the whole clonal fragment (i.e., the younger ramet plus the older ramet), stolon connection had no significant effect on any of the six growth measures in *W. chinensis*, but significantly increased all of them in *W. trilobata* (**Figure 6**; **Appendix Table 4**). Total biomass, leaf biomass, stem biomass, total stem length and total leaf area of the clonal fragment were larger in *W. trilobata* than in *W. chinensis* (**Figure 6**; **Appendix Table 4**).

## Discussion

Clonal integration can confer invasive alien clonal plants a competitive advantage over native ones under heterogeneous environments consisting of favorable and unfavorable patches (Wang et al., 2017). We found that clonal integration could also benefit the invasive plant *W. trilobata* more than that of its native congener *W. chinensis* heterogeneous environments with reciprocal patchiness of light and soil nutrients. However, the invasive species did not show a large ability of division of labor in such an environment.

The ability of plants to capture and utilize resources is an important determinant of their growth and fitness (Alpert, 1999; Feng et al., 2007b; Huang et al., 2018). To increase resources capture, plants are able to adjust the morphology and physiology of their roots and leaves, as well as biomass allocation to roots vs. to shoots, in response to the availability of above- and belowground resources (Burns and Winn, 2006; Magyar et al., 2007; Song et al., 2010; Roiloa et al., 2013; Keser et al., 2014). For non-clonal plants or isolated ramets, such responses are commonly in the way that is potentially helpful for them to increase the capture of the limiting resources (Bloom et al., 1985; Feng et al., 2007a; Roiloa et al., 2007). For connected ramets growing in heterogeneous environments, however, clonal integration may modify the responses of ramets so that they can respond in the way that is potentially helpful for them to increase the capture of the abundant resources (Stuefer, 1998; Ikegami et al., 2008; Wang et al., 2011; Roiloa et al., 2014; Huang et al., 2018).

Such specialization for abundance was observed in the younger ramet of the invasive plant *W. trilobata* growing in the high-light but low-nutrient patch in terms of photosynthetic physiology (i.e., greater net photosynthesis rate and chlorophyll content with than without clonal integration; **Figure 2**) and also in terms of biomass allocation (i.e., smaller root to shoot ratio with than without clonal integration; **Figure 4**). These physiological and allocational responses of the younger ramet can potentially increase its efficiency to take up light (Ikegami et al., 2008; Wang et al., 2011; Roiloa et al., 2014; Roiloa et al., 2016; Xi et al., 2019), which was abundant in the patch where the younger ramet grew. Specialization for abundance was also observed in the older ramet of *W. trilobata* growing in the low-light but high-nutrient patch in terms of root morphology (i.e., greater specific root surface area, total root length and total root surface area in the presence vs. absence of clonal integration) to increase nutrient capture (**Figures 3C, E, F**). These results suggest that, in the environment with reciprocal patchiness of light and soil nutrients, the interconnected ramets of the invasive plant *W. trilobata* demonstrated division of labor. Similarly, the interconnected ramets of the native plant *W. chinensis* also demonstrated division of labor in terms of root morphology of the older ramet (**Figures 3C–F**) and biomass allocation of the younger ramet (**Figure 4**).

Division of labor was also reported in many other clonal plants, including the invader *Carpobrotus edulis* and the native *Fragaris chiloensis* when their connected ramets grew in heterogenous environments with reciprocal patchiness of light and soil nutrients (Friedman and Alpert, 1991; Roiloa et al., 2007; Roiloa et al., 2014). The invader *Mikania micrantha* and the native plant *Trifolium repens* when their connected ramets grew in heterogenous environments with reciprocal patchiness of light and soil water (Stueffer et al., 1996; Huang et al., 2018). Additionally, connected ramets of *C. edulis* from both the native and invaded regions showed division of labor in terms of morphology and physiology (Roiloa et al., 2016) and connected ramets collected from dunes showed a greater capacity of division of labor than those from grasslands (Roiloa et al., 2007).

While the younger ramet of *W. chinensis* did not show specialization for abundance in terms of photosynthesis physiology (**Figure 2**), specialization for abundance in terms of biomass allocation of the younger ramet and specific root surface area and specific root length of the older ramet were significantly stronger in *W. chinensis* than in *W. trilobata* (**Figure 3**). Thus, in the heterogeneous environment consisting of reciprocal patches of light and nutrients, the overall ability of division of labor was not stronger in the invasive species *W. trilobata* than in the native one *W. chinensis*. The results also suggest that different species may show division of labor in terms of different sets of traits (morphological, physiological and allocational traits), as reported before (Roiloa et al., 2007; Wang et al., 2011; Roiloa et al., 2016; Huang et al., 2018; Xi et al., 2019).

In heterogeneous environments with reciprocal patchiness of resources, division of labor can commonly increase resource harvesting for ramets in both types of patches and consequently promote the growth of the whole clone (Roiloa et al., 2007; Roiloa et al., 2014). We indeed observed that, when connected ramets of the clonal invader *W. trilobata* grew in the heterogeneous environment with reciprocal patchiness of light and soil nutrients, clonal integration significantly increased the growth of both types of ramets and also promoted that of the whole clone (**Figures 5**, **6**). Surprisingly, however, this growth promotion was not observed in the native plant *W. chinensis*, despite the fact that clonal integration also resulted in division of labor in this species. These results suggest that division of labor may not always result in growth promotion, as its induction may incur greater costs (Alpert and Stuefer, 1997; Stuefer, 1998). The results also suggest at the first time that clonal integration can benefit invasive clonal species more than native ones when they grow in heterogeneous environments consisting of reciprocal patches of resources.

We conclude that in heterogeneous environments consisting of reciprocal patches of resources, which are common in natural habitats (Alpert and Mooney, 1996; Stuefer, 1998; Liao et al., 2003; Chu et al., 2006), clonal integration can confer invasive plants a competitive advantage over natives, but this difference is not related to their capacity of labor division. One caveat is that we used only one pair of invasive and native plant species so that the generality of the findings is limited. Further studies could consider using multiple species pairs to test the generality of our findings. Additionally, roles of clonal integration and division of labor in mediating competition between invasive and native clonal plants should also be tested. This study highlights the importance of clonal integration for plants in heterogeneous environments consisting of reciprocal patches of resources and suggests that clonal integration can contribute to the invasion success of alien clonal plants (Song et al., 2013; Wang et al., 2017; Roiloa et al., 2019).

## Data availability statement

The raw data supporting the conclusions of this article will be made available by the authors, without undue reservation.

## Author contributions

J-SC and L-XH contributed to conception and design of the study. L-XH, X-MZ, and XX conducted the experiments. L-XH, J-PL, and N-FL performed the statistical analysis. L-XH, F-HY, and MT wrote the first draft of the manuscript. All authors contributed to manuscript revision, read, and approved the submitted version.

## Funding

This research was funded by NSFC (grant 32071527).

## Acknowledgments

We gratefully thank Min Su and Hao Wang for assistance with the experiment.

## Conflict of interest

The authors declare that the research was conducted in the absence of any commercial or financial relationships that could be construed as a potential conflict of interest.

## Publisher’s note

All claims expressed in this article are solely those of the authors and do not necessarily represent those of their affiliated organizations, or those of the publisher, the editors and the reviewers. Any product that may be evaluated in this article, or claim that may be made by its manufacturer, is not guaranteed or endorsed by the publisher.
