# Supplementary material for: Clonal integration benefits an invader in heterogeneous environments with reciprocal patchiness of resources, but not its native congener
Source: Front Plant Sci. 2022 Dec 1;13:1080674. doi: 10.3389/fpls.2022.1080674 (PMC9751628; doi:10.3389/fpls.2022.1080674)
Supplement: Supplementary file 1 [file DataSheet_1.docx]

**Supplementary materials**

**Appendix Table 1** Two-way ANOVA results for effects of species, stolon connection and their interaction on the photosynthesis rate (P_n_) and chlorophyll content of the younger ramet. Values of *P* < 0.05 are in bold.

| Variable | Species (S) | |  | Stolon connection (C) | |  | S×C | |
| --- | --- | --- | --- | --- | --- | --- | --- | --- |
|  | *F*_1,28_ | *P* |  | *F*_1,28_ | *P* |  | *F*_1,28_ | *P* |
| P_n_ | 167.39 | **<0.001** |  | 7.59 | **0.011** |  | 2.61 | 0.119 |
| Chlorophyll content | 3.67 | 0.066 |  | 9.84 | **0.004** |  | 0.31 | 0.583 |

**Appendix Table 2** Two-way ANOVA results for effects of species, stolon connection and their interaction on morphology of (A) the younger and (B) the older ramet. Values of *P* < 0.05 are in bold.

| Variable | | | | | Species (S) | | | | | |  | Connection (C) | |  | S × C | |
| --- | --- | --- | --- | --- | --- | --- | --- | --- | --- | --- | --- | --- | --- | --- | --- | --- |
|  | | | | | *F*_1,28_ | | | | | *P* |  | *F*_1,28_ | *P* |  | *F*_1,28_ | *P* |
| (A) Younger ramet | | | | |  | | | | |  |  |  |  |  |  |  |
| Specific leaf area | | | | | 4.91 | | | | | **0.038** |  | 0.80 | 0.381 |  | 0.21 | 0.648 |
| Specific root length | | | | | 2.19 | | | | | 0.154 |  | 1.56 | 0.226 |  | 1.07 | 0.312 |
| Specific root surface area | | | | | 1.99 | | | | | 0.173 |  | 0.06 | 0.811 |  | 0.74 | 0.398 |
| Area per leaf | | | | | 42.31 | | | | | **<0.001** |  | 0.04 | 0.843 |  | 0.11 | 0.740 |
| Total root length | | | | | 3.54 | | | | | 0.074 |  | 7.12 | **0.014** |  | 0.22 | 0.641 |
| Total root surface area | | | | | 2.21 | | | | | 0.152 |  | 8.74 | **0.008** |  | 0.87 | 0.360 |
|  |  |  |  |  | |  |  |  |  |  |  |  |  |  |  |  |
| (B) Older ramet |  |  |  |  | |  |  |  |  |  |  |  |  |  |  |  |
| Specific leaf area | | | | | 2.82 | | | | | 0.108 |  | 7.87 | **0.011** |  | 5.39 | **0.030** |
| Specific root length | | | | | 2.49 | | | | | 0.130 |  | 11.35 | **0.003** |  | 1.53 | 0.230 |
| Specific root surface area | | | | | 2.35 | | | | | 0.140 |  | 23.18 | **<0.001** |  | 4.73 | **0.041** |
| Area per leaf | | | | | 13.58 | | | | | **0.001** |  | 1.37 | 0.256 |  | 7.64 | **0.012** |
| Total root length | | | | | 0.09 | | | | | 0.771 |  | 111.75 | **<0.001** |  | 0.02 | 0.877 |
| Total root surface area | | | | | 8.21 | | | | | **0.011** |  | 75.61 | **<0.001** |  | 2.22 | 0.156 |

**Appendix Table 3** Two-way ANOVA results for effects of species, stolon connection and their interaction on growth and allocation of (A) the younger and (B) the older ramet. Values of *P* < 0.05 are in bold.

| Variable | Species (S) | |  | Stolon connection (C) | |  | S×C | |
| --- | --- | --- | --- | --- | --- | --- | --- | --- |
|  | *F*_1,28_ | *P* |  | *F*_1,28_ | *P* |  | *F*_1,28_ | *P* |
| 1. Younger ramet |  |  |  |  |  |  |  |  |
| Total biomass | 136.75 | **<0.001** |  | 41.09 | **<0.001** |  | 39.96 | **<0.001** |
| Leaf biomass | 74.74 | **<0.001** |  | 30.60 | **<0.001** |  | 30.22 | **<0.001** |
| Stem biomass | 40.42 | **<0.001** |  | 2.54 | 0.128 |  | 0.67 | 0.424 |
| Root biomass | 1.58 | 0.225 |  | 2.88 | 0.108 |  | 3.89 | 0.065 |
| Total stem length | 31.69 | **<0.001** |  | 33.71 | **<0.001** |  | 26.70 | **<0.001** |
| Total leaf area | 156.56 | **<0.001** |  | 61.69 | **<0.001** |  | 66.80 | **<0.001** |
| Root to shoot ratio | 42.91 | **<0.001** |  | 46.18 | **<0.001** |  | 11.08 | **0.003** |
|  |  |  |  |  |  |  |  |  |
| 1. Older ramet |  |  |  |  |  |  |  |  |
| Total biomass | 177.07 | **<0.001** |  | 116.95 | **<0.001** |  | 64.49 | **<0.001** |
| Leaf biomass | 74.91 | **<0.001** |  | 53.38 | **<0.001** |  | 35.00 | **<0.001** |
| Stem biomass | 231.26 | **<0.001** |  | 123.52 | **<0.001** |  | 67.54 | **<0.001** |
| Root biomass | 4.21 | 0.056 |  | 17.68 | **0.001** |  | 2.40 | 0.140 |
| Total stem length | 21.51 | **<0.001** |  | 14.86 | **0.001** |  | 11.73 | **0.003** |
| Total leaf area | 80.37 | **<0.001** |  | 43.37 | **<0.001** |  | 45.96 | **<0.001** |
| Root to shoot ratio | 11.82 | **0.003** |  | 0.31 | 0.585 |  | <0.01 | 0.987 |

**Appendix Table 4** Two-way ANOVA results for effects of species, stolon connection and their interaction on growth of the whole fragment. Values of *P* < 0.05 are in bold.

| Variable | Species (S) | |  | Stolon connection (C) | |  | S×C | |
| --- | --- | --- | --- | --- | --- | --- | --- | --- |
|  | *F*_1,28_ | *P* |  | *F*_1,28_ | *P* |  | *F*_1,28_ | *P* |
| Total biomass | 197.15 | **<0.001** |  | 80.33 | **<0.001** |  | 62.27 | **<0.001** |
| Leaf biomass | 194.41 | **<0.001** |  | 100.84 | **<0.001** |  | 88.46 | **<0.001** |
| Stem biomass | 142.74 | **<0.001** |  | 44.23 | **<0.001** |  | 25.23 | **<0.001** |
| Root biomass | 4.29 | 0.054 |  | 0.98 | 0.336 |  | 5.50 | **0.031** |
| Total stem length | 30.04 | **<0.001** |  | 25.74 | **<0.001** |  | 20.36 | **<0.001** |
| Total leaf area | 97.55 | **<0.001** |  | 44.16 | **<0.001** |  | 47.19 | **<0.001** |
